# The Maintenance of Voluntary Hospitals

**Published:** 1923-03

**Authors:** H. G. James

**Affiliations:** Chairman Kent and Canterbury Hospital Propaganda Committee. 62 Bargate Street, Canterbury


### The Maintenance of Voluntary Hospitals.

SIR,—The maintenance of hospitals is a problem which must engage constantly the attention of the general public, and especially of those who are concerned in administration. A scheme has recently been introduced into East Kent which shows how adequate financial support may be obtained without recourse either to State or Rate aid. Briefly it is this. Those who wish to support the Kent and Canterbury Hospital undertake to deposit in a box (supplied by the Hospital Propaganda Committee) a sum averaging not less than 2d. per week, and to persuade other members of the family to make such contributions as they may be disposed, on which the hospital authorities admit to free treatment as in- or out-patients, boxholders and their dependents. A local representative of the Propaganda Committee is responsible for the collection every quarter of the moneys deposited, and is authorised to issue a certificate to the effect that the bearer is a contributor to the fund, and is a proper person on financial grounds to receive the benefits of the hospital.

The scheme has been in operation for nearly three years, and the moneys contributed have increased every year. In 1922 no less a sum than £4,800 was provided by this means—between one-half and two-thirds of the cost of maintaining the hospital apart from endowments. The scheme is in operation in about 100 villages and small country towns, and in spite of the fall in wages and the continuance of high prices, interest continues to increase. The fact of the matter is that the weekly contributors are primarily interested on religious and humanitarian grounds in the support of the hospital, for they recognise that it is a matter of Christian obligation. Should the need arise they take advantage of the benefits provided; but they do not regard their contributions in the light of "insurance" (for the words "health insurance" are not very popular in this part of England) but rather a gift to help those in need—in which category they or their families may or may not sometime be included. The truth is that the old Friendly Society spirit, so prevalent in the Foresters and in the Oddfellows, 30 and 40 years ago, dominates our subscribers; they "are out" to give and not to get.

Should your readers desire further information, I shall be happy to supply it.

H. G. James, Major,
Chairman Kent and Canterbury
Hospital Propaganda Committee.

62 Bargate Street,
Canterbury.

